# In Situ Following Oriented Crystallization of Pre-Stretched Poly(ethylene 2,5-Furandicarboxylate) Under Post Heating

**DOI:** 10.3390/polym17111508

**Published:** 2025-05-28

**Authors:** Jianguo Zhao, Mengcheng Yang, Binhang Wu, Hang Li, Yiguo Li

**Affiliations:** School of Materials Science and Chemical Engineering, Ningbo University, Ningbo 315211, China; z1102082022@163.com (J.Z.);

**Keywords:** poly(ethylene 2,5-furandicarboxylate) (PEF), stretching or tension, oriented crystallization and stress-induced crystallization (SIC), heat treatment, biobased polymers

## Abstract

Post-processing plays a vital role in the determination of the final structures and properties of oriented materials. As a sustainable candidate of oil-based poly(ethylene terephthalate), biobased poly(ethylene 2,5-furandicarboxylate) (PEF) reflects great promise in green fiber, film, and packaging applications, but it undergoes poor stress-induced crystallization (SIC) under tensile deformation, necessitating a post-processing technique to improve its crystallinity and stability. Here, the structural evolution of pre-stretched PEF under post heating after uniaxial deformation was monitored by online synchrotron X-ray diffraction/scattering, differential scanning calorimetry, and ex situ infrared spectroscopy. The results delineate the significantly enhanced crystallization of pre-deformed PEF that happened far below its cold crystallization temperature. Through the isochronous analyses of the temperature-dependent evolution of mechanical response, the mesophase, crystal structure, orientation factor, chain conformation, and interchain ═C−H···O═C hydrogen bonding, the molecular mechanisms of microstructural transition and oriented crystallization of pre-drawn PEF under post heating were clarified. This research can enhance the understanding of PEF crystallization in an oriented state and provide guidelines on the structural design and technical control for processing high-performance PEF-based materials.

## 1. Introduction

As an ideal and sustainable alternative to petrol-based poly(ethylene terephthalate) (PET), biomass-derived poly(ethylene 2,5-furandicarboxylate) (PEF) shows great promise in green film, fiber, and packing applications and thus has attracted the widespread attention and massive interest of both the academic and industrial communities [1,2,3,4,5]. In spite of its comparable heat stability, mechanical performance, and excellent gas barrier properties compared to PET, PEF exhibits an intrinsic drawback of poor crystallizability and slow crystallization rate [6,7,8,9], leading to the amorphous state formed upon common processing such as injection and blow moulding, concomitantly the rather poor thermal–mechanical performance of PEF-based materials [1,2,3]. To promote the crystallization of furan–polyesters, especially for PEF, several methods, such as copolymerizing with soft segments or adding plasticizers to improve its chain flexibility [10,11,12,13,14] and introducing various nanofillers as the nucleation agents to facilitate its nucleation [15,16,17,18,19,20,21,22,23,24], have been explored. Although certain enhancing effects have been found, past studies have focused on statistical crystallization, and the current enhancing level is still far from the demands of industrial manufacturers.

Generally, in industrialized processes, such as extrusion, drawing, blow molding, and mold pressing, the complex coupling of the stress and temperature field also facilitates the orientation and then crystallization, known as stress-induced crystallization (SIC), of long-chain semicrystalline polymers [25,26,27,28,29]. For example, uniaxially tensile deformation can trigger the SIC of natural rubber at room temperatures that are far above its crystallization temperature [25,26,27], and it also causes the SIC of PET and poly(lactic acid) (PLA) at temperatures near or even below their glass temperatures [27,28,29,30]. However, SIC usually exhibits a poor crystalline order that coexists with both an intermediate phase and internal stress [31,32,33,34]. To relax inner stress and further enhance crystallization, heat-setting or annealing is again an important processing technology of oriented products such as fiber and film, especially for poorly crystallized polymers like PET [28,35,36,37] and PLA [38,39,40]. It is, therefore, also of great interest and industrial significance to explore the structural evolution and enhanced crystallization of polymers in oriented states for tailoring their structures and properties.

For emerging PEF, current studies emphasize the structural behavior and SIC under uniaxial deformation. In 2018, Stoclet et al. first studied the structural evolution of amorphous PEF upon uniaxial drawing and discovered that its SIC occurred after a mesophase that cannot act as a precursor for SIC [41], differing from PET with a mesophase as an SIC precursor [33], and they found that the SIC of PEF showed a different crystalline structure from thermal-induced PEF. Meanwhile, Mao et al. reported the local ordering of amorphous chains with a preferred orientation prior to SIC [42]. In contrast, Forestier et al. found that the thermos-activated and stress-induced crystals of PEF reflected the same crystalline phase, and its SIC followed a simple two-step pathway without an intermediate phase [43,44,45,46,47,48,49]. They also approved of the idea that stretching reduces the number of activated interchain ═C−H···O═C hydrogen bonds [45], and they offered a proposal for improving the microstructural development and amorphous phase stability of SIC via the time/temperature superposition principle [47]. Relative to PET, the SIC of PEF is difficultly driven and only leads to a rather low crystallinity, necessitating post-processing to enhance crystallization and then to improve material properties.

However, to date, little attention has been paid to the structural evolution and oriented crystallization of deformed PEF under post-processing methods such as heat-setting and annealing. In addition, the heat-setting of oriented products like film and fiber in industrial production line is often performed by the rapid transfer of semi-manufactured products into the equipment at temperatures that are much higher than the one used for drawing. The course is completed within tens of seconds, so the structural development behavior is hardly followed. Therefore, the underlying microstructural transition and enhanced crystallization mechanisms even for well-studied PET are still poorly understood. Herein, after uniaxial deformation into a fixed strain, the structural development and oriented crystallization of pre-stretched PEF under post heating were followed by on-line synchrotron X-ray diffraction/scattering (WAXD/SAXS) combined with ex situ infrared (IR) spectroscopy and differential scanning calorimetry (DSC). After isochronous analysis of the temperature-dependent evolution of the mechanical relaxation, mesophase, crystal structure, orientation factor, conformation changes, and intermolecular ═C−H···O═C hydrogen bonding, molecular mechanisms of microstructural transformation and enhanced oriented crystallization of pre-deformed PEF in the post heating process were finally illuminated. This work deepens the understanding of microstructural behavior and enhanced crystallization of pre-oriented PEF, and thus provides guidelines on the structural design for processing high-performance PEF-based materials.

## 2. Materials and Methods

The granular PEF used in this work was kindly supplied by Ningbo Institute of Materials Technology & Engineering, Chinese Academy of Sciences, which has an intrinsic viscosity of 0.68 dL/g, a viscosity-average molecular weight of 22.0 kg/mol, and a glass transition temperature (*T*_g_), and melting point (*T*_m_) of 85, and 215 °C, respectively [22]. Dumbbell-shaped PEF samples of 50 mm × 4 mm × 1 mm were fabricated via microinjection molding using HAAKE Mini Jet II (Thermo Scientific, Waltham, MA, USA), as formerly described [22].

Uniaxial pre-stretching of PEF into a strain of 1.2 under 95 °C, and post heating with this unvaried strain to 150 °C, below its cold crystallization temperature (*T*_c_) of 162 °C [22], were performed on a Linkam MFS350 tensile stage (Linkam, Manchester, UK) by using temperature control equipment. A moderate strain rate of 0.1/min for pre-drawing was reached with a tensile speed of 25 μm/s, as the initial distance between the two clamps was 15 mm. A mild heating rate of 10 °C/min was employed for post heating. In addition, the engineering stress was also instantaneously recorded by the control software during the whole process.

Structural development was followed in situ by the WAXD/SAXS combined platform at the BL16B1 beamline of the Shanghai Synchrotron Radiation Facility in China, with an X-ray wavelength of 0.124 nm. Two-dimensional (2D) WAXD/SAXS images were captured simultaneously by utilizing the Pilatus 900 K and 2 M detectors with sample-to-detector distances of 220.6 and 2203.0 mm for the diffraction and scattering modes, respectively. The exposure and data acquisition times are 11.99, and 0.01 s, respectively. Infrared spectra were collected using a Nicolet 6700 FTIR spectrometer (Thermo Scientific, Waltham, MA, USA) equipped with the germanium crystal attenuated total reflection accessory at the BL16B1 beamline of the National Facility for Protein Science in Chain, and 32 scans were recorded in each acquisition at a resolution of 4 cm^−1^, and a testing range of 400~4000 cm^−1^. A single DSC heating scan of 30~260 °C at 10 °C/min was measured by a DSC 3+ (Mettler, Greifensee, Switzerland) within a nitrogen flow to further analyze the SIC under tension and post heating conditions.

## 3. Results and Discussion

Uniaxially tensile deformation of as-injected PEF was performed at a temperature (*T*_d_) of 95 °C above 10 °C of its *T*_g_ of 85 °C [22], which was recommended by Guigo et al. as the temperature is beneficial to develop the SIC of PEF [49]. After stretching into the pre-strain of 1.2, i.e., the later stage of strain-hardening before break at about the strain of 1.4 [49], post heating of as-drawn PEF constrained with this constant strain was implemented at a rate of 10 °C/min from 95 to 150 °C to investigate the influence of pre-stretching upon subsequent structural development and oriented crystallization in the post-processing course.

### 3.1. Simultaneously Mechanical Response

**Figure 1:**
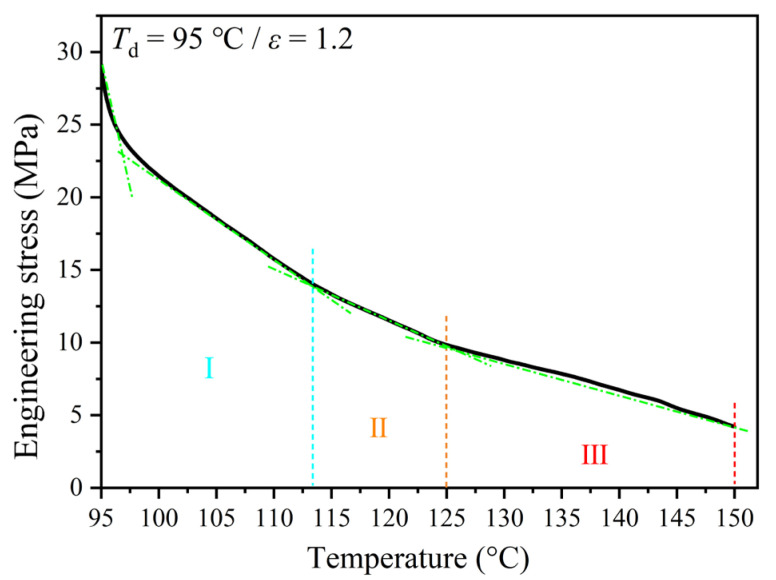
Mechanical relaxation of pre-stretched PEF having a constrained and constant strain of 1.2 during the post heating process at 10 °C/min after uniaxially tensile deformation at 95 °C.

Before analyzing the structural behavior, the mechanical response of pre-deformed PEF constrained with the constant strain was first followed. Based on the variation in its slope, the mechanical behavior of engineering stress with increasing temperature in the post heating course can be mainly divided into three stages, as delineated in Figure 1. At first, it was noted that once the tension terminated, the stress relaxed by 20% immediately, going from 29 to 23 MPa, which is ignored in the following stages since no structural change was detectable in this short timescale. In the first stage (I), the stress rapidly declined by another 35% to 13 MPa with the rise in temperature going from 97 to 113 °C, while in the second stage (II), it decreased less rapidly, with only a 10% decrement to 10 MPa at 125 °C. In the last stage (III), the stress decreased more slowly to 4 MPa (i.e., 15%), with a rise in temperature to 150 °C. This difference in stress decrease should implicate the changed structural development of pre-oriented PEF under the post heating, which will be uncovered by on-line simultaneous WAXD/SAXS experiments.

### 3.2. On-Line WAXD/SAXS Observations

**Figure 2:**
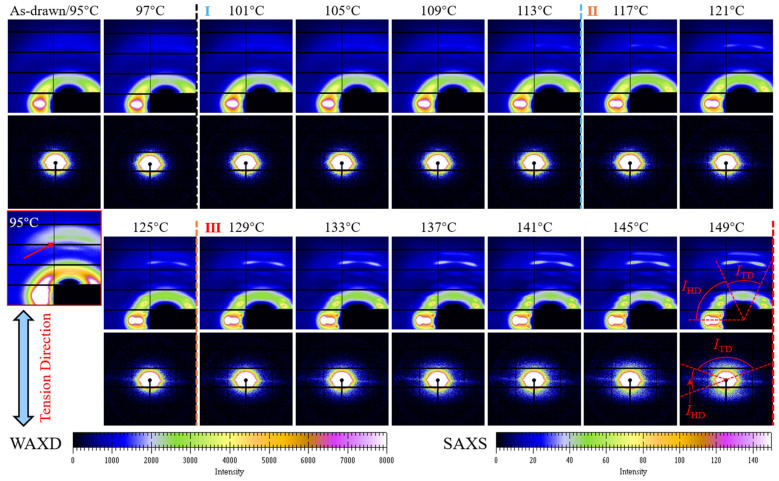
On-line 2D WAXD/SAXS images depicting the structural development in varied stages of pre-stretched PEF during the post heating process from *T*_d_ (95 °C) to 150 °C. To reflect the signal of mesophase, a red squared WAXD image of as-drawn PEF is also presented with an enlarged intensity contrast. The areas selected for intensity integration along the horizontal direction (HD) and vertical tension direction (TD) were also indicated in red in the last pair of WAXD/SAXS patterns.

Figure 2 exhibits time-resolved 2D WAXD/SAXS patterns with elevating temperature to delineate the structural evolution of pre-stretched PEF under post heating. For as-drawn PEF, despite the appearance of a highly centralized amorphous halo along the horizontal direction (HD), i.e., perpendicular to the vertical tension direction (TD), the rather poor diffraction signals in the WAXD image indicate that only trace strain-induced crystals formed even after late strain-hardening with a strain of 1.2. Concomitantly, a weakly equatorial SAXS streak normal for TD can be spotted, indicative of the existence of a fibril-like superstructure originating from either the long-chain bundles or fibrillar crystals [42]. These features affirm again the widely reported poor SIC of PEF [41,42,43,44,45,46,47,48,49]. As observed by Mao et al. [42], the local ordering in the axial direction is also verified by the second weak halo that appeared in TD, as highlighted by the inserted, red squared WAXD pattern. This behavior signifies the occurrence of a strain-induced mesophase [50]. Under post heating, clear diversities of structural evolution are evident in different stages, as predicted by stress analysis. In the sharp relaxation process, i.e., at 97 °C, no detectable change can be found in WAXD/SAXS images, and the termination of continued drawing should be responsible for the sudden decline of stress. In stage I, at 97~113 °C, an apparent shape change in the HD oriented amorphous halo was accompanied by the emergence of several diffraction points, and the TD signal of the mesophase also gradually became bright. However, the HD break of SAXS was almost unchanged. These phenomena should be attributed to the transformation of the highly oriented mesophase to fibrillar crystals, which is also in agreement with the rapid decrease in stress, since the oriented crystalline phase does not require tensile stress. These evolution features were accentuated with the rise in temperature from 113 °C to 125 °C, as confirmed by appearance of distinct diffractions in both the HD and TD. In addition, the TD mesophase was poorly evolved, and eventually disappeared, while the symmetrical scattering in the TD was detectable, although quite weak. The behavior indicates that besides a transition of the mesophase to fibrillar crystals, which further decreased stress by reducing the oriented amorphous content, oriented lamellae also started to develop [51]. That is, the synchronous generation of fibrillar crystals and oriented lamellae took place in stage II. Finally, the growth of oriented lamellae was dominated with a further rise in temperature from 125 to 150 °C in stage III, as approved by the continued intensifying of both the TD and HD diffractions as well as the symmetric TD scattering. Due to the limited transformation of oriented amorphous fractions into lamellar crystallization, the stress reduced more slowly.

**Figure 3:**
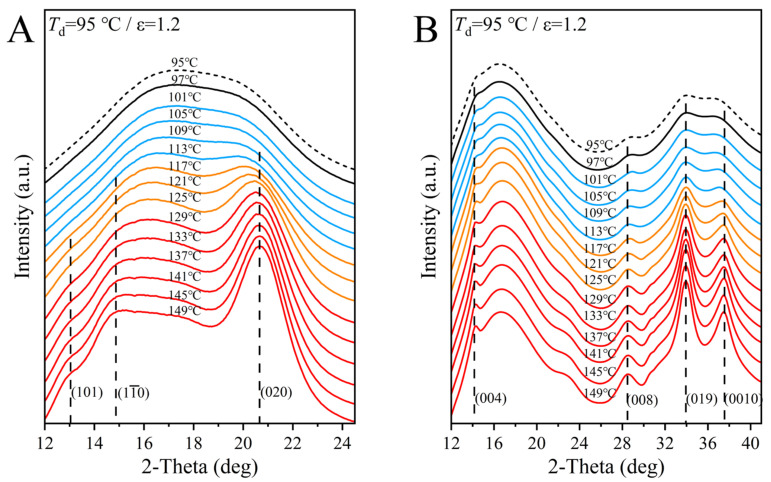
Temperature-dependent evolution of the integrated curves of 2D WAXD images in the (**A**) HD and (**B**) TD, respectively, and the integrated areas are indicated in the last pattern of Figure 2.

To further illustrate the structural evolution and oriented crystallization of pre-drawn PEF, Figure 3A,B show the WAXD curves integrated in the HD and TD, respectively. It is first emphasized that the diffraction patterns of Figure 2 show a high consistency with those of fiber diffractions, so that the crystal planes were indexed by the monoclinic crystal structure [52], as labeled in Figure 3. A comparison of the two profiles of as-drawn PEF at 95 °C illustrates that the SIC led to the higher order in the axial direction, as evidenced by the emergence of distinct (004), (008) and (0010) diffractions in the TD and the rather poor twice-connected broad peaks. This is unlike the more common single-bulge isotropic amorphous halo which appeared in the HD with the absence of separated diffraction, which is in accordance with the ordering mechanism proposed by Mao et al. [42]. Again, no distinguishable change is observed at 97 °C, and only slight enhancements of both the TD and HD peaks support the dominated transformation of the highly oriented mesophase to fibrillar crystals in stage I. Subsequently, the beginning growth of lamellar crystals in the transitory stage (II) resulted in the appearance of the characteristic (020) plane of the intermolecular order [52], and this structural development became dominated in the last stage (III). Herein, it has also been emphasized that in the last two stages, the diffraction intensities in both the TD and HD enhanced synchronously, supporting the synergistically inter- and intrachain orderings of lamellar crystallization in the lateral and axial directions, respectively. This differs from the fibrillar crystals showing a stress-induced preferential ordering in the axial direction. This characteristic will be further analyzed by the orientation factor in the following sections. Moreover, the perfection of fibrillar crystals should also be responsible for the enhanced (020) peaks in the latter two stages of II and III. Finally, it is also worth noticing that here, the oriented crystallization of pre-stretched PEF began at the temperatures much lower than the *T*_cc_ of the undrawn sample [22], which will be fully determined by the subsequent DSC measurements.

**Figure 4:**
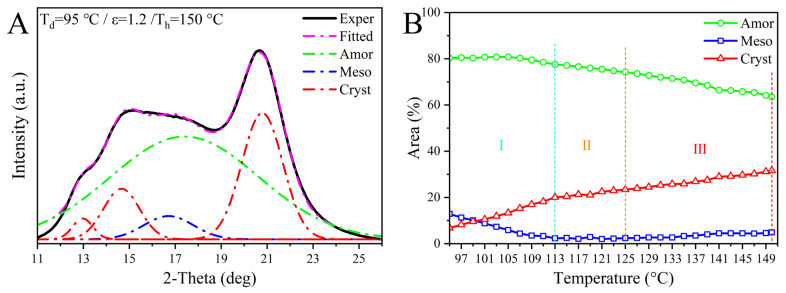
(**A**) An example for 150 °C showing a comparation of the experimental curve and the fitted areas of the amorphous, mesophase, and crystalline phases by Gaussian Function, and (**B**) evolution of the percentage areas of the three phases with elevating temperature in the post heating course.

Considering the coexistence of the amorphous, intermediate, and crystalline phases in pre-stretched PEF, the relative contents of the three phases were also fitted from WAXD curves via the Gaussian Function using PeakFit Software 4.12 [50]. Figure 4A shows a typical example at 150 °C when separating the areas of the three phases, and Figure 4B exhibits the temperature-dependent evolution of their percentage areas during the post heating course. The high consistency between the experimental and fitted curves, as illustrated in Figure 4A, supports the reliability of the fitting data [50]. As-drawn PEF shows a low percentage area of about 6%, in accord with a 5% crystallinity of SIC reported by Mao et al. at a similar tensile condition [42]. Upon heating, it is obvious that with the rise in temperature from 97 to 113 °C, the mesophase area decreases rapidly; in contrast, the content of the crystalline phase increases accompanied by an almost unvaried amorphous percentage, implying that the dominant transition of the mesophase to the crystalline phase happens in stage I. After a small decline in stage II, the mesophase ratio held a constant of nearly zero; instead, the area of amorphous phase slowly declined, leading to the continued enlargement of crystalline fraction, again supporting its transitory stage of co-occurrence of the change in the mesophase and amorphous phase into crystals. The increase in the crystalline phase was accentuated with rising temperatures from 125 to 150 °C, which is compensated by a synergetic reduction in the amorphous component, revealing that the dominated amorphous to crystalline transformation occurred in the last stage (III).

**Figure 5:**
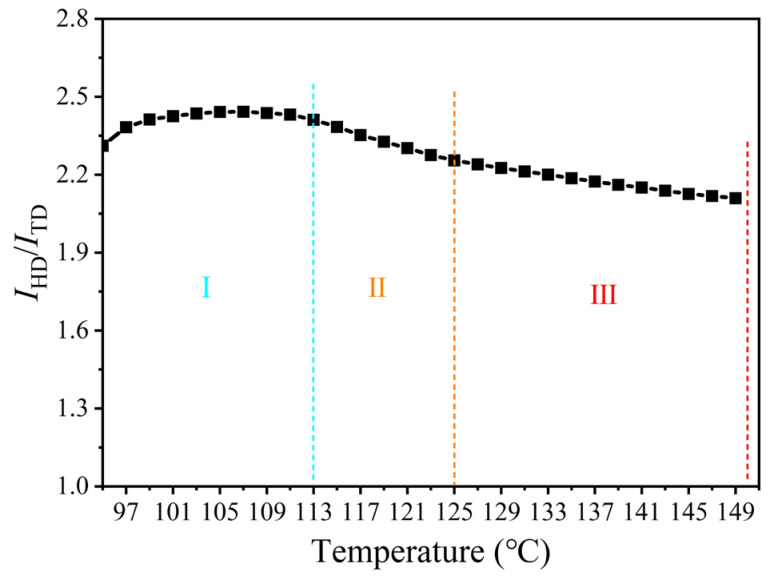
Temperature-dependent evolution of the relative scattering intensity *I*_HD_/*I*_TD_ along the HD and TD, respectively, during the post heating process of pre-stretched PEF with a fixed strain of 1.2.

Although the intensities of the HD streak and TD symmetric scattering are too weak to analyze the contents of fibrous and lamellar crystals, the ratio (*I*_HD_/*I*_TD_) of the integrated intensity in the HD and TD also offers insights to the oriented crystallization of pre-drawn PEF, as delineated in Figure 5. It is first apparent from the 2D SAXS images in Figure 2 that the HD scattering streak was always intensified, indicating that the growth and perfection of fibrillar crystals should proceed in the whole course, while the formation of lamellae began in stage II, and dominated in stage Ⅲ, as evidenced by the evolution features of the TD scattering. In stage I, the *I*_HD_/*I*_TD_ slightly enlarged, again demonstrating that the dominated fibrillar crystallization evolved from the mesophase, which decreased slowly in stage II and then rapidly in stage III; this is in line with the gradually dominated lamellar growth. It is emphasized that the *I*_HD_/*I*_TD_ ratio is always larger than one, implicating that the majority of fibrillar crystals were formed in good agreement with the area of the crystalline phase, which only increased from 20% at 113 °C during stage I, and to 30% at 150 °C during stage III (Figure 4B). A comparison of the poor diffractions (Figure 2) and the large *I*_HD_/*I*_TD_ at 113 °C further supports the continued perfection of fibrillar crystals throughout the whole course, since the SIC led to the much poorer crystalline order of PEF as compared to the thermal-induced crystallization [41,42,43,44,45,46,47,48,49].

**Figure 6:**
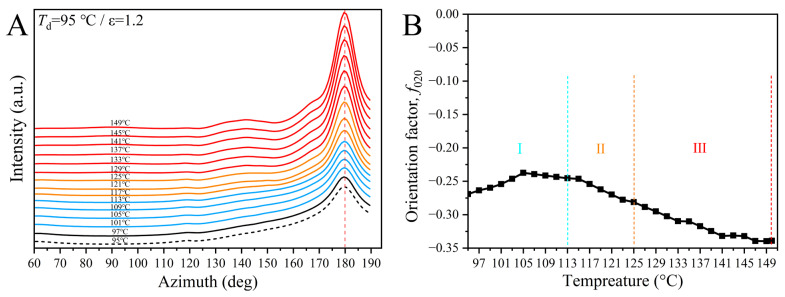
Temperature-dependent evolution of (**A**) the azimuthal intensity distribution of the (020) crystal plane and (**B**) the corresponding orientation factor, *f*_020_, under post heating. Here the *f*_020_ was calculated by using the position at 180°, as highlighted by the vertical dotted line in (**A**).

Orientation analysis is also informative. Figure 6 shows the changes in the azimuthal intensity distribution, and the corresponding orientation factor of the (020) crystal plane (*f*_020_) evaluated by the method proposed by Hermanns [53] as a function of temperature under post heating. The negative value of *f*_020_ is indicative of its perpendicular relationship with the TD. As-drawn PEF reflects a value of −0.27 at 95 °C, demonstrating that a high orientation of the crystals produced under SIC. The absolute *f*_020_ first declined in the early stage I, in conformity with the rapid stress relaxation (Figure 1), and then it became nearly unvaried, which may be ascribed to the formation of the network with a continued transition of the mesophase to crystalline phase that suppressing the further decrease in crystal orientation. Unexpectedly, the absolute *f*_020_ enlarged in the two following stages (II and III) with developing lamellar crystals. Two possible reasons could be responsible for this unusual phenomenon, for the (020) crystal phase is a reflection of the interchain order of crystalline PEF [52]. On the one hand, the continued perfection of stress-induced fibrillar crystals with an initially preferential axial order under post heating, as conformed by the synergetic enhancements of the diffractions and the HD scattering streak (Figure 2), not only improved the intermolecular order but eliminated the disturbance of the HD-oriented amorphous halo, leading to a low absolute *f*_020_ as calculated by the early poor (020) diffraction. On the other hand, here, the oriented crystallization of pre-stretched PEF took place at temperatures far below the *T*_cc_ of undeformed PEF (as unveiled in following DSC results), meaning that the crystalline ordering should be driven on the surface of highly oriented fibrillar crystals, i.e., via self-epitaxial nucleation, and grew from the oriented chains.

### 3.3. DSC Melting Measurement

**Figure 7:**
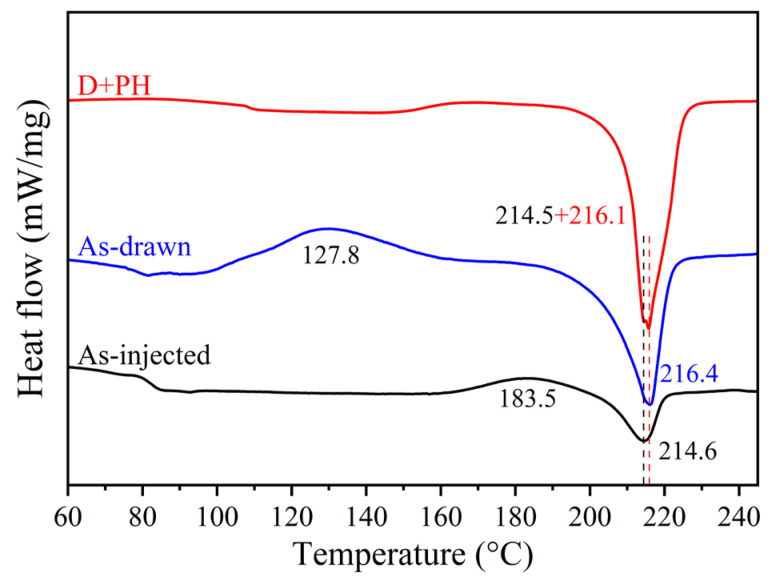
The first DSC heating profiles of the PEF samples processed under different conditions. Here the two dotted lines denotes the double melting peaks appeared in the D+PH sample.

To further understand the oriented crystallization of pre-deformed PEF, the DSC melting experiment was also performed at the same heating rate of 10 °C/min. Figure 7 displays the first heating profiles of as-drawn PEF with a strain of 1.2 and pre-stretched PEF after post heating (D + PH), with an as-injected sample as the reference; the corresponding thermal parameters of the three samples are listed in Table 1.

At first, the cold crystallization of as-injected PEF without tensile deformation occurred at temperatures, i.e., the onset and peak (*T*_cco_ and *T*_ccp_), of 163.8 and 183.5 °C, which are much higher than the final temperature of 150 °C for post heating. Moreover, the equal absolute values of cold crystallization enthalpy (Δ*H*_cc_) and heat of fusion (Δ*H*_m_) confirmed the amorphous structure of PEF after injection molding. After uniaxial drawing to a strain of 1.2, the cold crystallization of as-drawn PEF started at 105.9 °C (i.e., *T*_cco_) and reflected a peak temperature (*T*_ccp_) of 127.8 °C, which is in agreement with the on-line X-ray results presented in Figure 2. Meanwhile, the crystals showed a higher melting point (*T*_m_) of 216.4 °C than for (214.6 °C) undrawn PEF after cold crystallization. These phenomena further verify that enhanced crystallization of pre-stretched PEF should possess the nature of oriented crystallization. In particular, it shows an absolute crystallinity (Δ*X*) of 9.9%, i.e., the difference between Δ*X*_m_ and Δ*X*_cc_, which is higher than the area (6%) of the crystalline phase in Figure 4B, supporting that the mesophase and oriented amorphous components were attributed to the reduced enthalpy of oriented cold crystallization. As expected, cold crystallization is absent in the D + PH sample since the oriented crystallization of pre-drawn PEF has been finished upon post heating, and dual melting peaks are evident, which might originate from the lamellar and fibrillar crystals, respectively. In addition, a high crystallinity of 38.6% of the D + PH sample further corroborates the idea that the strongly enhanced crystallization of pre-stretched PEF occurred under post heating. It was also noticed that the absence of distinct dual melting peaks of as-drawn PEF might be influenced by the cooling to ambient conditions before the DSC melting experiment.

### 3.4. Ex Situ ATR-FTIR Analysis

Sousa et al. have affirmed that crystalline PEF has a zigzag pattern, i.e., the ethylene glycol (EG) moiety reflects a *trans* isomer and 2,5-furandicarboxylic acid (FDCA) fragment exhibits an energetically unfavorable *syn-syn* conformation (i.e., the two carbonyl oxygens pointing in the same direction as the furan-ring oxygen) that is locked by intermolecular ═C−H···O═C attractive interaction [55]. However, amorphous PEF chains favor a helical conformation of *gauche*^EG^ and *anti*^FDCA^ [55]. Hence, crystalline ordering of PEF should be completed synergically by the conformation changes in both EG and FDCA moieties, and the formation of highly ordered interchain ═C−H···O═C bonding. Tensile stress facilitated the conformation transition of linear macromolecules, favoring the SIC [26,27], but it reduced the activated hydrogen bonds between PEF segments [45]. It is, therefore, of high interest to compare the molecular orders of pre-stretched PEF before and after the post heating for further understanding of its oriented crystallization under post-processing.

**Figure 8:**
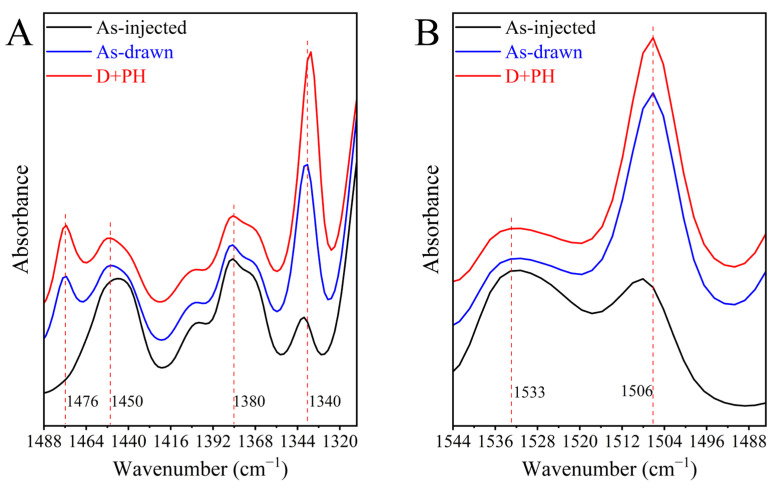
Infrared spectra of as-injected bar, as-drawn PEF, and pre-stretched PEF after post heating in the range of (**A**) 1488~1310 and (**B**) 1544~1484 cm^−1^, respectively.

Figure 8A,B show the infrared spectra of the three PEF samples in the range of 1488~1310 and 1544~1484 cm^−1^ to uncover the conformations of EG, and FDCA moieties, respectively. The strong bands at ~1450 cm^−1^ of the deformation and 1380 cm^−1^ of the wagging vibrations of CH_2_ groups demonstrate the dominated *gauche* EG segments in as-injected PEF, in line with its amorphous state [55]. After stretching, the signals of *trans* EG appeared at 1476 and 1340 cm^−1^ for both the CH_2_ deformation and wagging modes in as-drawn PEF, signifying the stress-induced *gauche* to *trans* transition of CH_2_ conformation that facilitated crystallization. After post heating, the wagging band of CH_2_ at 1340 cm^−1^ further intensified, approving the occurrence of enhanced oriented crystallization. Concomitantly, the *anti*^FDCA^ to *syn*^FDCA^ transformation is also supported by strong enlargement of the symmetric C═C stretching band at 1506 cm^−1^ after drawing (Figure 8B), which has been affirmed to be more sensitive to the conformation of FDCA fragment [56]. Unlike the further intensified 1340 cm^−1^ for aliphatic EG moiety upon post heating, the band intensity at 1506 cm^−1^ for aromatic *syn*^FDCA^ was almost unvaried, fully convincing that pre-drawing has finished the furan-ring flipping leading to the energetically unfavorable *anti*-to-*syn* transformation of FDCA segments, which is generally believed to be the restrictive step for PEF’s crystallization [57]. This should be the underlying reason for pre-drawn PEF having highly oriented chains which crystallize rapidly under post heating at the temperatures far below its *T*_cc_, as illustrated in Figure 2. However, the crystalline ordering of furanic polyesters with the energetically inaccessible *syn-syn*^FDCA^ conformation should be stabilized by well-organized interchain ═C−H···O═C hydrogen bonding [55,56], which is difficult to achieve under tensile stress [45].

**Figure 9:**
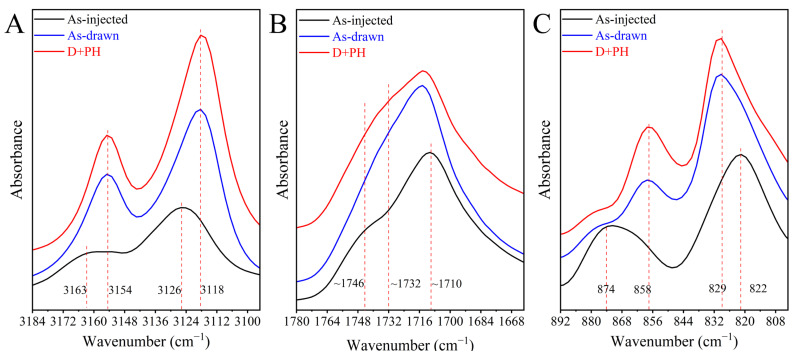
Infrared spectra of the as-injected bar, as-drawn PEF, and pre-stretched PEF after post heating in the range of (**A**) 3184~3094, (**B**) 1780~1662, and (**C**) 892~802 cm^−1^, respectively.

Forming intermolecular ═C−H···O═C hydrogen bonding reflects a high sensitivity to the vibrational modes of both the furanic ═C−H and C═O groups [23,45,55,56]. Figure 9A shows the infrared spectra in the 1544~1484 cm^−1^ range to describe the stretching of ═C−H for the three samples. The weak band at 3126 cm^−1^ for symmetric ═C−H stretching again identifies the amorphous structure of as-injected PEF without generating ═C−H···O═C bonding [55]. After tension, the apparent red-shift in the vibration from 3126 to 3118 cm^−1^ and intensity increasement of the latter imply the presence of intermolecular ═C−H···O═C bonding in as-drawn PEF. On the contrary, the sample shows the poor diffraction patterns, as depicted in Figure 2. Stretching decreases the intermolecular distance that should facilitate the interchain interaction [58]. However, the crystalline order of PEF should be achieved by formation of highly ordered ═C−H···O═C bonding that is limited upon stretching [45]. It is therefore suggested that poorly organized ═C−H···O═C bonding should exist in the pre-stretched sample, which can be well-organized under post heating, as evidenced by the further enhancement of the band at 3118 cm^−1^ in the D + PH sample. This again supports the simultaneous perfection of fibrillar crystals and growth of lamellae during the post heating process, as unveiled by in situ X-ray observations (Figure 2, Figure 3, Figure 4, Figure 5 and Figure 6). The C═O bands are difficult to assign because of the multiple contributions from free to H-bonded and from *anti*^FDCA^ to *syn*^FDCA^ conformers [45,55]. Even so, a new band at ~1732 cm^−1^, corresponding to the calculated wavenumber of H···O═C bonded C═O of *syn*^FDCA^ moiety [55], appears in as-drawn PEF, and its intensity increases after post heating, as presented in Figure 9B. In addition, we have also confirmed the formation of intermolecular ═C−H···O═C bonding leading to the same band at ~1730 cm^−1^ in crystalline poly(butylene 2,5-furandicarboxylate) [23,24]. It is, therefore, reasonable that the change in C═O vibrations again influences the two-step development of ═C−H···O═C interactions in PEF under drawing and post heating conditions. Finally, the change in the characteristic peak of the crystalline order at 858 cm^−1^ of the three samples again confirms the conclusions given above (Figure 9C).

### 3.5. Structure Evolution Mechanism

**Figure 10:**
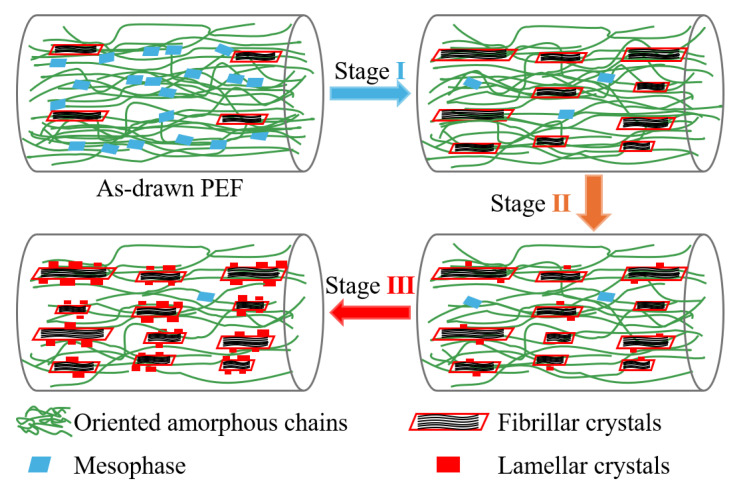
Schematic illustration of the microstructural development and oriented crystallization mechanism of pre-stretched PEF under post heating constrained with a fixed pre-strain.

According to the structural analyses given above, microstructural transformation and oriented crystallization mechanisms of pre-stretched PEF under post heating are schematically illuminated in Figure 10. In addition to generating trace stress-induced fibrillar crystals, tensile deformation at 95 °C into late strain hardening also led to the co-existence of the mesophase with an axial order and highly oriented amorphous chains in as-drawn PEF. Upon post heating constrained with the fixed pre-strain, the microstructure development follows the three stages, depending on the elevating temperature. In the first stage (I) of the low temperature range of 97~113 °C, the mesophase transformed into the crystalline phase, leading to the co-occurrence of enlarging initial SIC crystals and producing new fibrillar crystals. Concomitantly, the stress decreased rapidly. In the subsequent stage (II) of 113~125 °C, accompanied with the decline in the amount of mesophase and, inversely, the increase in fibrillar crystals, oriented lamellar crystals began to develop, and a mild mechanical relaxation was observed. These crystals can afford highly ordered crystalline surfaces which act as the template for driving self-epitaxial nucleation. As the temperature rose further to reach above 125 °C, the growth of the lamellae dominated the structural development in the last stage (Ⅲ), and the stress declined much more slowly. Meanwhile, as the temperature elevated, the perfection of the fibrillar crystals caused by SIC and the transition of mesophase at low temperatures took place throughout the whole process. In particular, the pre-stretching completed the process of furan-ring flipping from the low-energy, helical *gauche*^EG^*anti*^FDCA^ conformation, leading to an energetically unfavorable, zigzag pattern with the *trans*^EG^*syn*^FDCA^ geometry [55], which overcame the restrictive step for producing crystalline order of furan-aromatic polyesters [57]. Although it has been found that drawing reduced the number of activated hydrogen bonds since it suppressed the precise matching of ═C−H and C═O groups in intermolecular segments [45], stress relaxation of oriented chains with *syn*^FDCA^ conformers upon post heating promoted their relative displacement that facilitated the matching of ═C−H and C═O groups to activate the formation of intermolecular H-bonding, and thus drastically enhanced the crystallization of pre-drawn PEF with a highly oriented state at a temperature far below its *T*_cc_. Therefore, one can conclude that the enhanced crystallization of pre-drawn PEF under post heating should originate from a synergistic effect of stress-induced intramolecular conformation order along the axial direction and thermal-induced perfection of intermolecular hydrogen bonding in the lateral direction.

## 4. Conclusions

In summary, the oriented crystallization of pre-stretched PEF after drawing at 95 °C into late strain hardening with a strain of 1.2 under post heating from 95 to 150 °C was studied by in situ WAXD/SAXS combined with DSC and FTIR. We demonstrated that pre-tension led to a change in segment conformations from the helical geometry to an energetically unfavorable zigzag pattern. This was accompanied by the existence of the mesophase, which induced a drastically enhanced crystallization of pre-stretched PEF at temperatures far below the *T*_cc_ of undrawn PEF, leading to an enlargement in crystallinity from below 10% of the SIC to near 40% after post heating. Moreover, the oriented crystallization of pre-stretched PEF followed the three stages with elevating temperatures. In the first stage, the transformation of the stress-induced mesophase to crystalline phase, forming fibrillar crystals, dominated the structural development at temperatures below 113 °C; then, oriented lamellae began to develop on the surface of fibrillar crystals via self-epitaxial nucleation in the second transitory stage at 113~125 °C, and, finally, the growth of lamellar crystals became dominant in the last stage at 125~150 °C. Further, the perfection of stress-induced fibrillar crystals took place over the whole course. Although stretching reduced the number of activated intermolecular ═C−H···O═C bonding, leading to the poor SIC of PEF [45], the relaxation of oriented chains with *syn*^FDCA^ conformers that was induced by pre-stretching upon post heating promoted the relative displacement of ═C−H and C═O groups, which facilitated their precise matching to activate the formation of interchain H-bonding, leading to a drastically enhanced crystallization of pre-deformed PEF. The current work not only agrees that pre-drawing induced conformation transition, facilitating the oriented crystallization of pre-drawn PEF at low temperatures, but uncovers its underlying molecular mechanism via the coupling of the mesophase to crystal transformation and self-epaxial nucleation; therefore, it can guide the structural design for processing high-performance PEF-based materials.

## Figures and Tables

**Table 1:** Characteristic thermal parameters of three PEF samples with varied processing conditions.

Samples	*T*_cco_ (°C)	*T*_ccp_ (°C)	Δ*H*_cc_ (J/g)	*X*_cc_ (%) *	*T*_m_ (°C)	Δ*H*_m_ (J/g)	*X*_m_ (%) *	Δ*X* (%)
As-injected	163.8	183.5	−8.5	6.2	214.6	9.4	6.9	0.7
As-drawn	105.9	127.8	−30.6	22.3	216.4	44.2	32.3	9.9
D + PH	-	-	-	-	214.5/216.1	52.9	38.6	38.6

* *X*_cc_ and *X*_m_ are the ratios of the absolute values of **Δ***H*_cc_ and **Δ***H*_m_ with the equilibrium melting enthalpy of 100% crystalline PEF (${∆H}_{m}^{0}$) of 137 J/g [54].

## Data Availability

The original contributions presented in the study are included in the article, and further inquiries can be directed at the corresponding authors.
